# DEMMI Scores, Length of Stay, and 30-Day Readmission of Acute Geriatric Patients in Denmark: A Cross-Sectional Observational Study with Longitudinal Follow-Up

**DOI:** 10.3390/geriatrics4010008

**Published:** 2019-01-07

**Authors:** Dorte Melgaard, Maria Rodrigo-Domingo, Marianne M. Mørch, Stephanie M. Byrgesen

**Affiliations:** 1 Physio- and Occupational Therapy Department, North Denmark Regional Hospital, DK-9800 Hjørring, Denmark; s.byrgesen@rn.dk; 2 Center for Clinical Research, North Denmark Regional Hospital, DK-9800 Hjørring, Denmark; 3 Unit of Epidemiology and Biostatistics, Aalborg University Hospital, DK-9000 Aalborg, Denmark; mariarodrigo@rn.dk; 4 Aalborg University Hospital, Psychiatry, DK-9000 Aalborg, Denmark; 5 Geriatric Department, North Denmark Regional Hospital, DK-9800 Hjørring, Denmark; m.moerch@rn.dk

**Keywords:** mobility, elderly, De Morton Mobility Index, readmission, length of stay

## Abstract

The aims of this study are to describe the mobility of acute geriatric patients, the length of stay, and to characterise patients who were readmitted within 30 days based on the De Morton Mobility Index (DEMMI). A cross-sectional observational study with longitudinal follow-up was conducted in the period from 1 March 2016 to 31 August 2016. Inclusion criteria were acute geriatric patients hospitalised for a minimum of 24 h. Of the 418 patients hospitalised during the study period, 246 (59%) participated in this study (44% male, median age 83 years [70; 94]). For patients in an acute geriatric department, the median DEMMI score was 41 and the mean score was 39.95. Patients with a DEMMI score ≤40 show a significantly lower Barthel 100 index, lower 30 s. sit-to-stand scores and were significantly more likely to be bedridden or, amongst those not bedridden, to use a mobility aid. Lower DEMMI scores were associated with longer admissions. DEMMI seems to have the ability to predict discharge within one week. There was no significant association between a lower DEMMI score and higher risk for 30-day readmission. Further research is needed to determine whether the DEMMI is suitable for identifying the patient’s need for further rehabilitation following the discharge.

## 1. Introduction

The level of mobility is an important indicator of illness in the elderly and a strong prognostic factor of health in geriatric patients [1,2,3,4]. Loss of mobility can result in a loss of independence, increased reliance on care and caregivers, increased hospital admission, and increased economic expenses [5]. Furthermore, loss of mobility can lead to institutionalisation, sarcopenia secondary to immobilisation, poor quality of life, pneumonia, and mortality [6]. 

Mobility is defined by the International Classification of Functioning (ICF) as “moving by changing body position or location or by transferring from one place to another, by carrying, moving or manipulating an object, by walking, running, or climbing, and by using various forms of transportation” [7].

Daily activities related to mobility include moving from a bed to a chair, going to the restroom, climbing the stairs, cooking, shopping, and traveling. Limitations in mobility make it difficult for elderly people to be independent in their daily activities and to participate in social activities. Several studies have documented that limited mobility is a marker for risk of adverse outcomes, loss of independence, and institutionalisation [8,9,10,11]. 

Potential impairment can be identified by measuring mobility in the elderly. An accurate measure of mobility can also help clinicians to establish a baseline that can be followed by a reassessment. Difficulties related to performing daily activities, reasons for difficulty with specific tests, and possible health risks caused by immobility such as falls have to be included in mobility assessments in the elderly patient [12]. 

Several instruments exist to assess mobility limitations in elderly people. Some are self-reported, including the Environmental Analysis of Mobility Questionnaire, the Life-space Assessment, and the Rosow-Breslau Scale. Others are performance based, such as the 6 min. Walk Test, the Timed Up-and-Go test, the Berg Balance Test, or the De Morton Mobility Index (DEMMI) [13,14].

The DEMMI was developed to identify early signs of physical decline, assist with goal setting, monitor recovery, and, of course, prompt early intervention. The DEMMI consists of 15 hierarchical mobility items: movement in bed (3), chair (3), static balance (4), walking (2), and dynamic balance (3). An interval score ranging from 0–100, where 0 represents very poor mobility and 100 represents a high level of independent mobility, is scored by a physiotherapist [15]. The DEMMI was developed using the Rasch model and validated in older medical patients in an acute hospital setting in Australia [15,16]. Clinometric properties have been reported in patients with hip fracture, Parkinson’s disease, geriatric conditions, and in rehabilitation [17,18,19,20]. A study reporting normative data in a population of healthy, community-dwelling adults age 60+ documented a mean DEMMI score of 81 [14]. Another study describing a population of older acute medical patients documented that patients discharged to their homes had a mean DEMMI score of 62.14 (SD 18.41) and patients discharged to inpatient rehabilitation had a mean DEMMI score of 50.75 (SD 11.29) [21]. No studies have documented DEMMI as a screening tool for predicting readmission [22]. A Danish nationwide register-based cohort study documented that pre-hospital factors indicate the risk of readmission more than hospital factors [23].

In Denmark, the DEMMI score is information that needs to be reported to the National Danish Geriatric Database [24]. The physiotherapists that specialised in geriatric medicine in North Denmark Regional Hospital question whether DEMMI gives them useful knowledge when taking into account the time they spend on performing the test.

The aims of this study were to describe the mobility of acute geriatric patients using DEMMI, to describe the length of stay (LOS) and investigate potential associations to the DEMMI score, and to characterise patients’ readmission within 30 days of discharge and see whether readmission is related to the DEMMI score.

## 2. Materials and Methods 

**Figure 1:**
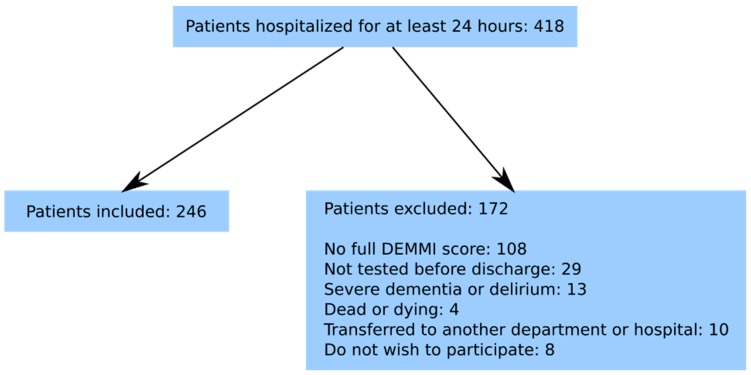
Flowchart of included patients.

From 1 March 2016 to 31 August 2016, a cross-sectional observational study with longitudinal follow-up was conducted. The Danish version of the DEMMI was during the study period used to test patients hospitalised in the Department of Geriatric Medicine at the North Denmark Regional Hospital [16]. Patients with stroke were admitted directly to the neurological department and are therefore not part of this study. Inclusion criteria were ≥60 years old and hospitalisation for a minimum of 24 h. Of the 418 patients hospitalised during the study period, 246 (59%) participated in our study. The present study was registered with the Danish Data Protection Authority (2008-58-0028). According to the local Ethics Committee written informed consent was not required. The exclusion criteria are illustrated in Figure 1.

Data were collected during the hospital stay on Barthel 100, 30 s. sit-to-stand test, Body Mass Index (BMI), waist circumference (2 cm above the navel), circumference of the lower leg (15 cm above the lower edge of the patella), and circumference of the upper arm (lateral epicondyle + 10 cm), and strength in the dominant hand. The physiotherapists performing DEMMI were experienced in the geriatric field and trained in testing with DEMMI. From the National Patient Register we obtained the patients’ age, gender, admission date, discharge date, Charlson Comorbidity Index (CCI), and acute readmission within 30 days of discharge. In the present study, readmission was defined as hospitalisation due to disease with which the patients were discharged in the Northern Region of Denmark.

### Statistical Analysis

Categorical demographic variables were summarised as the number and percentage of patients in each category, and continuous variables were summarised as their median (5% percentile; 95% percentile) or their mean (std.), depending on their distribution. The median DEMMI score of 41 was used as cut-off to classify patients as having a low (<41) or high (≥41) DEMMI score. To analyse the sensitivity of our conclusions to the cut-off choice, all analyses that include the dichotomised DEMMI variable were repeated setting 36 or 46 as the cut-off instead (sensitivity analyses). Associations between categorical variables were studied using Fisher’s exact test or the chi-squared test (as appropriate for the number of observations). Two-group comparisons of continuous variables were done using the non-parametric equality of the medians test.

We used linear regression to investigate a possible linear association between LOS and the DEMMI score. Besides this, the predictive accuracy of the DEMMI scores regarding discharge within one week (that is, LOS ≤ 7 days) and 30-day readmission was assessed by the area under the curve (AUC). We used bootstrapping to avoid overestimating the performance [25], this was done using the command *comproc* in Stata [26]. In these analyses, we used the original DEMMI scores instead of the dichotomised version defined above. Patients who died while admitted were not included in the “discharged within one week” analysis, nor were they or patients that died within 30 days of discharge without being readmitted included in these “readmitted within 30 days of discharge” analyses. Results with a *p*-value < 0.05 were considered statistically significant. We did a “complete case” analysis in each model, that is, we did not input data. Statistical analyses were performed using Stata Version 13.1 (Stata Corporation, College Station, TX, USA).

## 3. Results

**Figure 2:**
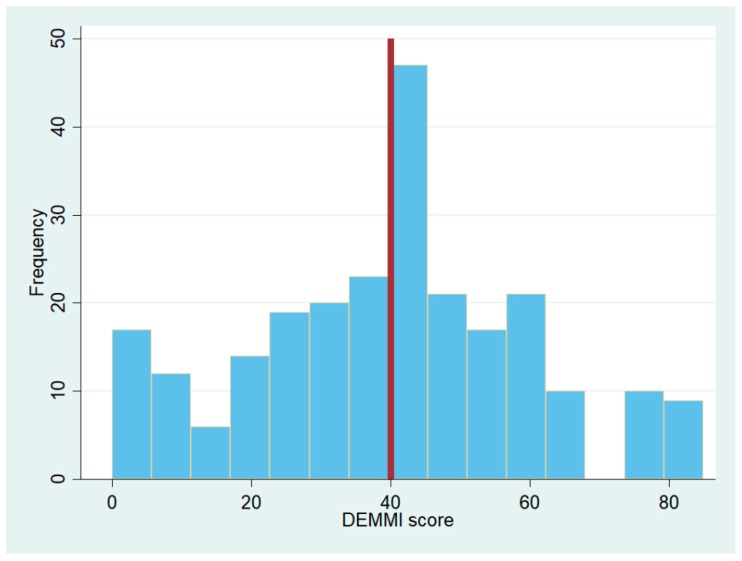
Histogram of DEMMI scores from patients in acute geriatric departments. The vertical line is set at the median DEMMI score in the group.

In total, 246 patients were included in the project (44% male, median age 83 years [70; 94]). The median DEMMI score for patients in an acute geriatric department was 41 [0; 74] as illustrated in Figure 2. The mean score was 39.95 (SD 20.59).

As illustrated in Table 1, we found significant differences between the two DEMMI groups regarding the reason for hospitalisation, their Barthel 100 index, and their 30 s. sit-to-stand test, the presence of dysphagia, the percentage of bedridden patients and, amongst those not bedridden, of patients using mobility aid, the length of admission, the existence of a rehabilitation plan for the patient, and, finally, their discharge location. Changing the cut-off to 35 (88 patients had a score equal to or below 35, 158 above) or to 45 (158 patients had a score equal to or below 45, 88 above) did not affect the conclusions. That is to say, although the percentages or median values for each variable changed in the low and high DEMMI score groups depending on the cut-off, the same variables remained significantly different between the high and low DEMMI score groups across all three cut-offs.

No statistically significant differences were found regarding the list of variables between the patients readmitted within 30 days and those not readmitted, as presented in Table 2. The prevalence of dysphagia in this study population is documented to be 50% and patients with dysphagia have a significant lower DEMMI score and a longer LOS [27]. 

**Figure 3:**
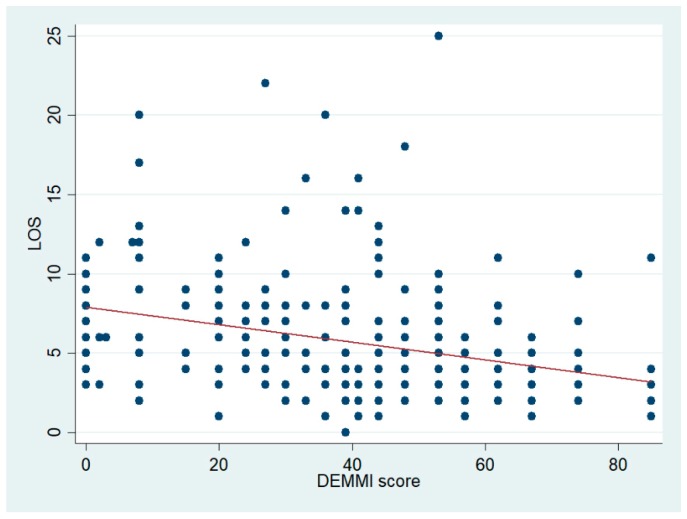
Scatter plot of LOS and the DEMMI score with the fitted regression line shown in red.

Figure 3 shows a significant negative association between DEMMI scores and LOS. Patients with lower DEMMI scores had longer LOS. The regression coefficient was −0.05 (95% CI [−0.07, −0.03]), although very little variation was explained (adjusted R^2^ 0.075), as can also be observed in the figure. This analysis was done using all patients. Removing patients who died during admission did not affect the estimated coefficients. 

The DEMMI score seems to have a reasonable predictive accuracy regarding discharge within one week. Indeed, the AUC was 0.668 (95% CI [0.587, 0.750]) when using bootstrap and, of course, was slightly higher when using the whole sample of 239 patients: 0.693 (95% CI [0.612, 0.774]. In contrast, the DEMMI score has a very low predictive accuracy regarding 30-day readmission, at least when looking at the AUC. In this case, the AUC when using bootstrap was 0.461 (95% CI [0.375, 0.548]), while it was a bit higher but still rather low: 0.509 (95% CI [0.421, 0.598]) when using the whole sample of 232 patients. We did not find any statistically significant differences between patients that were readmitted and those not readmitted in a large range of parameters, as shown in Table 2. 

## 4. Discussion

In this study, the patients were consecutively included and a full DEMMI test was performed in 59% of all admitted geriatric patients. A relatively high number of patients (26%) were excluded due to the fact only a partwise DEMMI test was performed. This fact seems relevant as it may indicate a limitation of patients where the physiotherapists are able to test with the DEMMI. The overall reasons for 41% of the acute geriatric patients not being tested with the DEMMI may be, for example, physiotherapists not working on weekends, patients sent for X-ray, or the patients are suffering from severe dementia or delirium or do not want to participate in the test. Other studies also demonstrate missing assessments with the DEMMI [28,29]. The patients were tested within 24 h after admission. The LOS is relatively short and if the DEMMI score should form the basis for the intervention of physiotherapists it has to be performed shortly after hospitalisation. The DEMMI is known as a valid and reliable assessment tool and is free from floor and ceiling effects [15,16,19,20].

There was a significant difference between patients with a DEMMI score ≤40 and >40 according to the reason for hospitalisation, Barthel 100, 30 s. chair-stand test, dysphagia, use of mobility aids, bedridden, admission time, rehabilitation plans and discharge destination. The mean DEMMI score of 40 reported in this study is arguably lower than the ones that were reported earlier. This is not surprising given the characteristics of our group of acute geriatric patients. In a study of healthy, community-dwelling adults 60+, the mean age was 74.6 years and the mean DEMMI score was 81.0. This study showed that patients’ older age had significantly lower DEMMI scores [14]. De Morton et al. demonstrate that acute medical patients who were discharged to inpatient rehabilitation had a DEMMI score of 50.75 versus patients discharged to home with a score of 62.14 [21]. A Danish study including medical patients 65+ years showed a median DEMMI score of 44 [28]. This patient group is somehow comparable with the patients included in the present study, and the DEMMI score is as well. Another Danish study including 369 patients (mean age 77.9 years) admitted in the emergency department and at a 30-day follow up demonstrated that 30 s. chair-stand test assessment at admission was able to identify mobility limitations 30 days after discharge [29]. In both Danish studies, the focus is on limitations in mobility and not on readmission. 

A study where DEMMI is performed in surgical patients (20–87 years), documents that the DEMMI score can predict discharge within 1 week [30]. The present study confirms these results in an acute geriatric population, although the predictive accuracy that we show is slightly lower. This might be because we have used bootstrap to avoid overestimating the performance [25], or because of the different natures of the populations. Their population was younger but also presented lower preoperative DEMMI scores than our patients. 

To the best of our knowledge, no other studies have focused on DEMMI as a tool to predict the risk of readmission. The present study showed that the DEMMI was unable to predict the risk of readmission in a group of acute geriatric patients. There were no significant differences between patients readmitted within 30 days and those not readmitted regarding parameters describing demographic characteristics or mobility.

Dichotomising a variable by splitting it at the median (also known as median split) is a common although controversial technique [31,32,33]. For this reason, we have run our analyses with DEMMI as both a dichotomous and a continuous variable. The cut-off of <41 that splits our group of patients into two was chosen as the median observed DEMMI score in an attempt to find a clinically relevant cut-off given that the ones suggested in the literature are too high to be relevant in our study. We investigated two other cut-offs shifting the original by 5 points up or down, which did not change which variables are statistically significantly different between the two DEMMI groups. 

According to our data, 80% of patients who were discharged to a nursing home had a low DEMMI score. This study did not include data about the living situation of the patients before hospitalisation. It is unknown whether the high percentage of discharge to nursing homes in the low DEMMI group was due to the fact the patients lived in a nursing home prior to hospitalisation or to a lower level of mobility after hospitalisation. Whether or not the DEMMI score can be useful as an indicator when choosing the appropriate discharge location is thus uncertain, while another focus for further research is whether the DEMMI is suitable for identifying the patient’s need for and effect of further rehabilitation after discharge.

## Figures and Tables

**Table 1:** Baseline demographics and clinical characteristics for the whole group and for the two subgroups defined after dichotomisation of the DEMMI score.

	N = 246	DEMMI ≤ 40 N = 111	DEMMI > 40 N = 135	*p*-Value
Gender (male) ^a^	109 (44%)	53 (48%)	56 (42%)	0.325
Age (years) ^b^	83 [70; 94]	83 [69; 95]	84 [70; 93]	0.435
Reason for hospitalisation ^a^				<0.001
Pneumonia	16 (7%)	7 (6%)	9 (7%)	
Dyspnea	28 (11%)	14 (13%)	14 (10%)	
Dehydration	15 (6%)	10 (9%)	5 (4%)	
Fall	27 (11%)	15 (14%)	12 (9%)	
Reduction in food intake	11 (5%)	9 (8%)	2 (1%)	
Infections	59 (24%)	28 (25%)	31 (23%)	
Diverse	65 (26%)	14 (13%)	51 (38%)	
Pain	25 (10%)	14 (13%)	11 (8%)	
Charlson Comorbidity Index ^b^	2 [0; 5]	2 [0; 5])	2 [0; 9])	0.938
Barthel 100 ^b^	67.5 [8; 94]	38 [0; 77]	79 [39; 95]	<0.001
Body Mass Index ^b^	25.4 [17.9; 37.2]	25.4 [19.1; 43.8]	25.6 [17.9; 34.3]	0.623
Weight (kg) ^b^	69 [45; 99.3]	67.5 [45.1; 102.4]	69.25 [45; 97.6]	0.847
Waist line (cm) ^b^	100 [75; 127]	100 [83; 136]	100.5 [74; 120]	0.909
Lower leg circumference (cm) ^b^	32 [26; 42]	32 [24; 42]	32 [26; 43]	0.891
Upper arm circumference (cm) ^b^	27 [21; 34]	28 [21; 36]	27 [20; 34]	0.144
Handgrip—dominant hand (kg) ^b^	18.9 [7.6; 40.4]	15.97 [5.6; 37]	19.87 [8.43; 44.63]	0.172
Sit-to-stand test (repetitions) ^b^	0 [0; 10]	0 [0; 0]	0 [0; 11]	<0.001
Dysphagia present ^a^	118 (50.0%)	66 (59.5%)	52 (38.5%)	0.001
Bedridden ^a^	22 (9.8%)	21 (21.2%)	1 (0.8%)	<0.001
Mobility aid ^a,^*	146 (72.3%)	73 (93.6%)	73 (58.9%)	<0.001
Admission time (days) ^b^	5 [2; 11]	6 [2; 14]	4 [1; 11]	<0.001
Rehabilitation plans ^a^	186 (75.6%)	96 (86.5%)	90 (66.7%)	<0.001
Discharged to ^a^				
Own home	155 (63%)	47 (42%)	108 (80%)	
Nursing home	20 (8%)	16 (14%)	4 (3%)	
Rehabilitation	38 (16%)	31 (28%)	7 (5%)	
Unknown	33 (13%)	17 (16%)	16 (12%)	<0.001

^a^ Number (%). ^b^ Median [5% quantile; 95% quantile]. * Only computed for those not bedridden. It includes rollator, crutches, and wheelchair.

**Table 2:** Characteristics of patients readmitted or not within 30 days from discharge.

	Readmitted within 30 Days after Discharge N = 52	Not Readmitted within 30 Days after Discharge N = 180	*p*-Value
Gender (male) ^a^	21 (40.4%)	82 (45.6%)	0.509
Age (years) ^b^ DEMMI score ^c^	85.0 [69.0; 93.0] 40.58 (19.85)	83.0 [70.0; 94.0] 41.28 (20.66)	0.529 0.827
Reason for hospitalization ^a^			0.329
Pneumonia	2 (3.8%)	14 (7.8%)	
Dyspnea	8 (15.4%)	17 (9.4%)	
Dehydration	5 (9.6%)	8 (4.4%)	
Fall	4 (7.7%)	22 (12.2%)	
Reduction in food intake	2 (3.8%)	7 (3.9%)	
Infections	8 (15.4%)	46 (25.6%)	
Diverse	15 (28.8%)	49 (27.2%)	
Pain	8 (15.4%)	17 (9.4%)	
Charlson Comorbidity Index ^b^	2 [0; 7]	2 [0; 5]	0.446
Barthel 100 ^b^	76 [0; 100]	68 [8; 90]	0.736
Body Mass Index ^b^	23.5 [18.0; 37.6]	25.7 [17.8; 37.2]	0.261
Weight (kg) ^b^	67.7 [47.0; 92.1]	70.5 [42.8; 101.0]	0.462
Waist line (cm) ^b^	96.5 [75.0; 135.0]	102.0 [75.0; 126.0]	0.127
Lower leg circumference (cm) ^b^	32.0 [24.0; 39.0]	33.0 [26.0; 43.0]	0.273
Upper arm circumference (cm) ^b^	26.0 [19.0; 34.0]	27.0 [21.0; 36.0]	0.897
Handgrip—dominant hand (kg) ^b^	20.0 [5.9; 40.4]	18.8 [8.6; 44.6]	0.673
Sit-to-stand test (repetitions) ^b^	0 [0; 9]	0 [0; 10]	0.731
Dysphagia present ^a^	23 (44.2%)	82 (45.6%)	0.866
Bedridden ^a^	3 (6.1%)	16 (9.5%)	0.459
Mobility aid use ^a,^*	36 (78.3%)	108 (71.1%)	0.336
Length of stay in hospital (days) ^b^	5 [1; 16]	4 [2; 12.5]	0.208
Plans for rehabilitation ^a^	44 (84.6%)	132 (73.3%)	0.094
Discharged to ^b^			0.303
Own home	36 (69.2%)	117 (65%)	
Nursing home	1 (1.9%)	17 (94%)	
Rehabilitation	8 (15.4%)	28 (15.6%)	
Unknown	7 (13.5%)	18 (10.0%)	

^a^ Number (%). ^b^ Median [5% quantile; 95% quantile]. ^c^ Mean (SD). * Only computed for those not bedridden; it includes rollator, crutches, and wheelchair. This table does not include the seven patients who died during admission or the seven patients who died within the 30-day period without being readmitted.
